# Estimating Patient-Level Uncertainty in Seizure Detection Using Group-Specific Out-of-Distribution Detection Technique

**DOI:** 10.3390/s23208375

**Published:** 2023-10-10

**Authors:** Sheng Wong, Anj Simmons, Jessica Rivera Villicana, Scott Barnett

**Affiliations:** 1 Applied Artificial Intelligence Institute, Deakin University, Burwood, VIC 3125, Australia; 2 School of Computing Technologies, RMIT University, Melbourne, VIC 3000, Australia

**Keywords:** EEG, rule-based reasoning, uncertainty estimation, out-of-distribution, seizure detection

## Abstract

Epilepsy is a chronic neurological disorder affecting around 1% of the global population, characterized by recurrent epileptic seizures. Accurate diagnosis and treatment are crucial for reducing mortality rates. Recent advancements in machine learning (ML) algorithms have shown potential in aiding clinicians with seizure detection in electroencephalography (EEG) data. However, these algorithms face significant challenges due to the patient-specific variability in seizure patterns and the limited availability of high-quality EEG data for training, causing erratic predictions. These erratic predictions are harmful, especially for high-stake domains in healthcare, negatively affecting patients. Therefore, ensuring safety in AI is of the utmost importance. In this study, we propose a novel ensemble method for uncertainty quantification to identify patients with low-confidence predictions in ML-based seizure detection algorithms. Our approach aims to mitigate high-risk predictions in previously unseen seizure patients, thereby enhancing the robustness of existing seizure detection algorithms. Additionally, our method can be implemented with most of the deep learning (DL) models. We evaluated the proposed method against established uncertainty detection techniques, demonstrating its effectiveness in identifying patients for whom the model’s predictions are less certain. Our proposed method managed to achieve 87%, 89% and 75% in accuracy, specificity and sensitivity, respectively. This study represents a novel attempt to improve the reliability and robustness of DL algorithms in the domain of seizure detection. This study underscores the value of integrating uncertainty quantification into ML algorithms for seizure detection, offering clinicians a practical tool to gauge the applicability of ML models for individual patients.

## 1. Introduction

Epilepsy is a chronic neurological disorder that affects close to 1% of the population worldwide. It is characterized by recurrent and unpredictable brain activities, known as epileptic seizures [1,2]. Patients suffering from epileptic seizures face a 2–3 times higher mortality rate. Hence, correctly diagnosing and controlling epileptic seizures is of the utmost importance to reduce the mortality rate in patients suffering from epilepsy.

An electroencephalography (EEG) test records the electrical activity in the cerebral cortex via the electrodes that are placed on top of the scalp of the patients [2,3,4]. Seizure detection in EEG data is an important aspect of the diagnosis and management of epilepsy. By reviewing and interpreting the recorded EEG data, clinicians can look for abnormal EEG patterns that are indicative of epileptic seizures, leading to an accurate diagnosis and treatment. Nevertheless, this process of EEG analysis is labor-intensive and open to subjective interpretation, potentially introducing bias [5,6,7].

In recent years, machine learning (ML) algorithms have been proposed as a way to assist clinicians in annotating and reviewing EEG recordings by identifying potential seizure segments [8,9,10,11,12,13,14,15]. It works by highlighting potential segments of interest that may be an indication of seizures, reducing the time and effort needed by clinicians to review large amounts of EEG recordings. This positively impacts the patient’s outcome by allowing for rapid diagnosis and treatment.

Multiple studies have shown its ability to detect seizures with an accuracy of over 90% [9,11,13,16,17,18,19,20,21]. While these algorithms show promise, they face multiple challenges in being able to consistently detect seizures across all patients suffering from different types of seizures. One of the many challenges in developing robust seizure detection tools is the variability in seizure patterns across different seizure types, which makes it difficult to identify a consistent pattern for training machine learning algorithms [2,22,23]. Further, patients suffering from various seizure types might exhibit a range of distinct EEG patterns, making it hard for ML algorithms to detect them if it has not been exposed to the patterns being evaluated during the training process. This challenge is further exacerbated by the limited availability of high-quality EEG data for training due to stringent data privacy concerns and regulations, which can restrict the algorithm’s ability to generalize across diverse patient groups [24]. As a result, ML algorithms for seizure detection may struggle to accurately detect seizures in patients whose seizure patterns were not represented in the training data.

This can lead to uncertain and unreliable predictions when the algorithms encounter unfamiliar EEG patterns from certain patient groups. This lack of confidence can be especially dangerous when seizure detection algorithms are deployed in real time, where the cost of a misdiagnosis is high [25,26]. For example, incorrect predictions can lead to over-diagnosis, resulting in unnecessary medical interventions, or under-diagnosis, causing missed seizures that can have severe consequences for patients such as permanent injuries or death. To improve the robustness of seizure detection tools and address the challenges posed by the lack of reliability prediction of ML algorithms in some patients, it is essential to develop effective methods that warn users of patients where their ML models are not confident in their predictions to ensure the patient’s safety and care.

In this study, we propose a novel method that leverages concepts from the field of rule-based approach, out-of-distribution (OOD) detection [27] and uncertainty quantification [28] to identify high-risk patients for whom the model has low confidence in its predictions. Unlike most out-of-distribution (OOD) detection and conventional uncertainty estimation approaches, which typically focus on individual data points, our approach considers a set of records of EEG data corresponding to a patient. This shift in focus offers a more realistic and practical approach. Uncertainty in individual data points, in a dataset that could contain millions of such data points, does not provide meaningful information to clinicians. A patient-level analysis, on the other hand, gives a holistic view of the patient’s condition over time, which is far more useful in a real-world clinical setting.

Departing from traditional methods, which often directly classify uncertainty or OOD data based on raw EEG data points, our approach harnesses the internal features representation of a DL model and Deep Support Vector Data Description (Deep SVDD) to learn what the model has successfully learned and areas where it falters. Deep SVDD is an unsupervised learning method designed for detecting out-of-distribution, abnormal data [29]. It leverages neural networks together with the SVDD to learn low-dimensional representations of input data in a latent space. Inspired by the support-vector machine (SVM), the Deep SVDD approach captures the distribution of the input data by projecting it onto a hypersphere in the latent space that minimizes distances. This hypersphere is then used to determine whether data points are out-of-distribution.

This patient-level analysis enables a nuanced understanding of the model’s prediction confidence for a patient. By detecting these low-confidence patients, our approach aims to reduce the occurrence of high-risk predictions in unseen seizure patients and improve the robustness of the existing seizure detection algorithms. Another key strength of our approach is its flexibility where it is compatible with any DL architecture, which potentially broadens its applicability and utility in various EEG scenarios. To the best of our knowledge, this study represents the first attempt to leverage these techniques to enhance the robustness and reliability of ML algorithms in the field of seizure detection.

This novel approach where we utilized and combined existing techniques, could be integrated into an existing seizure detection pipeline to identify patients unsafe for DL predictions in clinical settings. This approach mitigates the risk of incorrect or missed diagnoses when clinicians rely on DL algorithms for insights. By alerting clinicians to patients with high prediction uncertainty, it allows clinicians to manually oversee the process of seizure detection and annotation process, thereby enhancing the outcome of patients. This is especially useful when DL models face issues of generalizability and robustness.

## 2. Related Works

Uncertainty estimation allows users to quantify the confidence associated with a model’s predictions, thereby enabling the identification of potentially unreliable predictions. The uncertainty in a model can originate from various factors, including a mismatch between training and testing data, the model’s learning capacity and the presence of noise in the training data. Popular techniques for uncertainty estimation encompass Monte Carlo Dropout (MC-Dropout) and Bayesian Neural Networks (BNNs) [30,31].

Conversely, out-of-distribution (OOD) detection involves recognizing data that significantly diverge from the training data distribution [32]. It enables users to be alerted to new or anomalous training data patterns. This ‘out-of-distribution’ data stems from distinct distributions and may be triggered by a shift in the data distribution, insufficient training data or variations in data collection methodologies, among other factors. Common OOD detection methods include maximum SoftMax probabilities, distance-based methods and SVDD [27,32,33,34].

Both uncertainty estimation and OOD detection contribute to the model’s ability to generate robust and reliable predictions. Consequently, they equip users to make informed and secure decisions. These techniques are particularly beneficial in high-risk applications such as healthcare, where prediction reliability and the ability to handle anomalies can significantly impact the well-being of the patients. It is also important to note that many studies on OOD detection and uncertainty estimation often develop and use their own unique methods to solve their problems that are domain specific.

Despite advancements in applying state-of-the-art machine learning models, such as DL for EEG-based seizure detection, there is a lack of research focusing on uncertainty estimation and OOD detection specifically in the context of EEG-based seizure detection. This gap in the literature presents a significant challenge in the clinical adoption of seizure detection algorithms. Most existing research tends to prioritize the improvement of model performance, focusing predominantly on statistical metrics such as accuracy, without adequately addressing the crucial issues of uncertainty or the presence of OOD data. We will focus on explaining techniques applied in the field of general healthcare.

The most common uncertainty estimation technique, MC-Dropout, is frequently used in conjunction with DL. Multiple studies in the field of healthcare have applied MC-Dropout to improve their reliability alongside their predictions. For instance, one study applied MC-Dropout to their proposed Shallow Convolutional Neural Network (SCNN-MCD) for motor imagery classification in patients with severe disabilities [35]. Another study used MC-Dropout with their DL model, DeepSleepNet-Lite, for sleep-scoring prediction uncertainty estimation [36].

The Bayesian Neural Network (BNN) is a DL model that estimates uncertainty based on the predictions. Instead of single-value estimates for each weight in a deterministic neural network, BNN learns a probability distribution for each weight, where different values of weights can be sampled [31]. This gives the BNN the ability to produce a range of outputs, enabling the ability to estimate uncertainty. One study, for instance, used BNNs to detect epileptogenic brain malformations, achieving a 5% better accuracy compared to non-Bayesian learners using the same network architecture [37]. Another study proposed using the BNN along with confidence calibration to improve the estimating uncertainty in classifying five-class polyps from colonoscopy [38]. The proposed BNN with confidence calibration beat state-of-the-art algorithms at close to 80% accuracy after rejecting samples with a high uncertainty.

Another study utilized conformal predictions to estimate uncertainty in histopathological diagnoses [39]. This uncertainty estimation method offers a prediction interval to identify unreliable predictions, achieving only 2% errors compared to 25% without the method. Another group of researchers proposed the use of predictive entropy for the classification of myocardial infarction using ECG, successfully detecting uncertainty in predictions made by their DL model [40].

Researchers leveraged a modified version of the SVDD technique to detect abnormal living patterns in nine elderly individuals using infrared motion sensors [41]. The goal was to provide effective patient monitoring for individuals living alone. This method achieved an impressive average accuracy of 95.8% in detecting abnormal patterns.

Another study evaluated various OOD methods on multiple medical image datasets on their ability to reject images unseen by the models [42]. They found that no single OOD method consistently outperformed the others across all medical image datasets. However, a binary classifier with feature representation from the penultimate layer and the Mahalanobis distance-based method demonstrated superior performance on average across all datasets.

In conclusion, existing techniques for uncertainty estimation and OOD detection, largely designed and tested for other domains in healthcare, may not be fully suited to the unique challenges posed by EEG seizure data. The complexity and variability of EEG signals require tailored methodologies for effective uncertainty estimation and OOD detection. Additionally, frequently employed methods like SoftMax confidence or logit confidence scoring are overly simplistic and insufficient for addressing the unique requirements of this field. They fall short when applied to EEG-based seizure detection, as revealed in subsequent experimental stages. Further, in reviewing the current literature on uncertainty estimation and OOD detection techniques, it becomes apparent that many proposed methodologies are specifically crafted for their respective fields and may not transition effectively to other domains.

To bridge this gap, we introduce a novel method specifically designed for EEG-based seizure detection. Utilizing the internal representations of a DL model and Deep SVDD, our approach delivers a comprehensive patient-level analysis, thus providing a more efficacious solution for uncertainty detection at the patient level in seizure detection tasks.

## 3. Materials and Methods

### 3.1. Data Description and Acquisition

We utilized The Children’s Hospital Boston Massachusetts Institute of Technology (CHB-MIT) dataset, which comprises 916 h of scalp EEG data from 23 pediatric patients consisting of 5 males and 17 females aged from 3 to 22 suffering from intractable seizures [43,44]. The publicly available EEG dataset is popular and widely used for the development and evaluation of seizure detection algorithms [12]. Many DL models have been developed and evaluated using the CHB-MIT dataset [13,45,46,47,48,49]. This dataset consists of 664 EEG recordings of mostly 1 h long, and some longer EEG recordings, with a sampling rate of 256 Hz. Compared to other EEG datasets, this dataset stands out for its long-term continuous EEG recordings (>12 h) with minimal disruptions, ideal for the development of seizure detection systems [24].

Each EEG recording is paired with annotations of the precise onset and end times of seizure events. In total, there are 198 seizures recorded in the dataset, spread across all patients. Each patient has various number channels following a 10–20 electrode placement method, consisting of 23 to 26 channels. The dataset contains different seizure types, such as focal, lateral and generalized seizures.

### 3.2. Preprocessing

Before feeding the EEG signals from the recordings into the model for training, several preprocessing steps were undertaken. To ensure consistency in the number of channels across different recordings and patients, 22 EEG channels were selected, eliminating any duplicate channels, channels unique to a single patient and non-EEG channels. The EEG data are normalized to a range of 0–1. Additionally, a Finite Impulse response (FIR) bandpass filter with a range of 1–60 Hz and the removal of the DC component were implemented to reduce noise in the EEG data. The FIR bandpass filter was also essential to isolate relevant EEG signals associated with seizure events in the Delta, Theta, Alpha, Beta and Gamma sub-bands [2].

**Figure 1:**
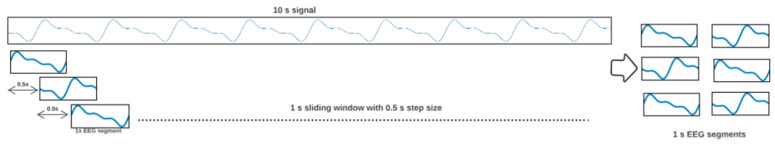
Shows the segmentation of EEG signals of a single channel into multiple 1 s sliding window with 0.5 s step size. This process is repeated for every channel in the EEG recordings.

Patients suffering from epileptic seizures often exhibit normal brain activity, the majority of them only exhibit abnormal EEG patterns during a seizure or right before a seizure and spend less than 1% of their time in a seizure stage. Hence, the EEG recordings are often highly imbalanced. Given the imbalanced nature of the EEG dataset, with the majority class being interictal, a sliding window method with a step size of 0.5 was employed on segments containing seizure activity to increase the sample data representing seizures. This method is commonly used in seizure detection to increase the sample size of EEG recordings [13,17,49]. For each given seizure segment, the sliding window was moved in steps that resulted in 50% overlap between consecutive segments. This overlap technique effectively doubled the seizure segments derived from the same seizure recordings. A visual representation can be seen in Figure 1. Each EEG recording was split into one second EEG segments, where EEG segments were randomly sampled at a ratio of 5:1 to reduce the data imbalance. Further, in order to ensure the model is able to learn effectively, we included preictal and postictal EEG data, where 30 s of EEG segments before a seizure and 30 s of EEG segments after a seizure were included as part of the data used. In order to preserve the sampling information time, no further signal transformation or features engineering took place.

### 3.3. Estimating Patient-Level Uncertainty

**Figure 2:**
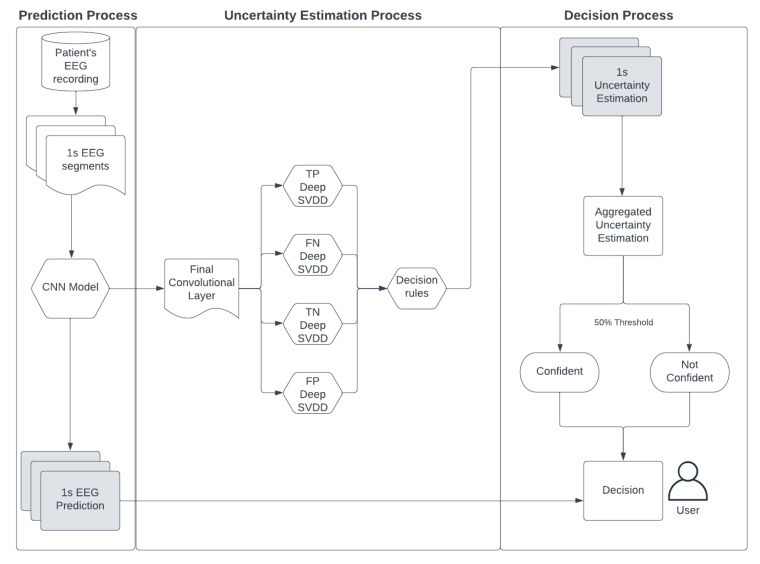
Shows the process during inference time. The patient’s EEG recordings are divided into 1 second segments for seizure detection and uncertainty estimation simultaneously. Two results will be produced, the predictions and uncertainty score where the users will then make a final decision.

In summary, the proposed method tries to determine the uncertainty of a Convolutional Neural Network (CNN) model’s predictions for a patient. Using an unsupervised learning technique (Deep SVDD) to learn the distribution of each of the four groups for true positives (TP), false positives (FP), true negatives (TN) and false negatives (FN), all of which are derived from the model’s training predictions. This categorization, guided by a rule-based approach, refines the prediction possibilities for each EEG data segment during inference. This process is applied to each segment of the provided EEG data. The algorithm then aggregates the binary uncertainty predictions for these segments, resulting in a value between ‘0′ and ‘1′, which indicates the model’s confidence level. The uncertainty score generated by our proposed method enables users to decide on whether to trust the predictions made by our DL model for a specific patient. Our proposed method, which encapsulates the entire uncertainty estimation process, is visually represented in Figure 2 (inference).

To understand the trained model’s capabilities, we input the training EEG data into the trained CNN model for inference. This facilitated the segregation of the EEG samples into the 4 aforementioned groups. This categorization offers us a valuable proxy of the training patterns that the model learned and those that it fails to learn. For example, the TP and TN groups contain samples that the model correctly predicted, indicating patterns that the model has successfully learned, whereas the FP and FN groups encapsulate patterns that the model has not effectively learned.

In the subsequent step, we extracted the values obtained from the internal representations (final convolutional layer) of the trained CNN model for each sample in each of the groups (TP, TN, FP, FN). These extracted values were then utilized to train four distinct Deep SVDD models—one for each group. The role of these Deep SVDD models, each being a three-layered neural network fused with the SVDD model, is to learn and understand the distribution of patterns specific to their respective group. The outputs produced by the Deep SVDD are binary scores, where ‘0′ signifies that the input segment is in-distribution (pertaining to that particular group), while ‘1’ indicates out-of-distribution.

A set of rules is devised to estimate uncertainty based on the predictions from the models. The CNN models produce either a prediction of ‘0′ (no seizures) or ‘1′ (seizures). If the prediction is ‘0′ (non-seizure) and either the FN or TN group prediction is ‘0′ (in-distribution), while the FP and TP predictions are ‘1′ (out-of-distribution), it suggests that the model is likely confident in its non-seizure prediction. This is because some groups are associated with “non-seizure” patterns—the FN group contains examples where the model failed to identify “non-seizure” patterns, and the TN group includes those where the model correctly learned these patterns. Therefore, if either the FN or TN group aligns with the model’s “non-seizure” prediction, it suggests that the identified EEG pattern corresponds to a typical “non-seizure” case, either one that the model usually identifies correctly or one that it often misses. On the other hand, the FP and TP groups are associated with seizure segments. As such, when the model predicts ‘0′ (non-seizure), it would be contradictory for the EEG segment to belong to these seizure groups. Hence, reinforcing the model’s confidence for prediction of non-seizure.

**Figure 3:**
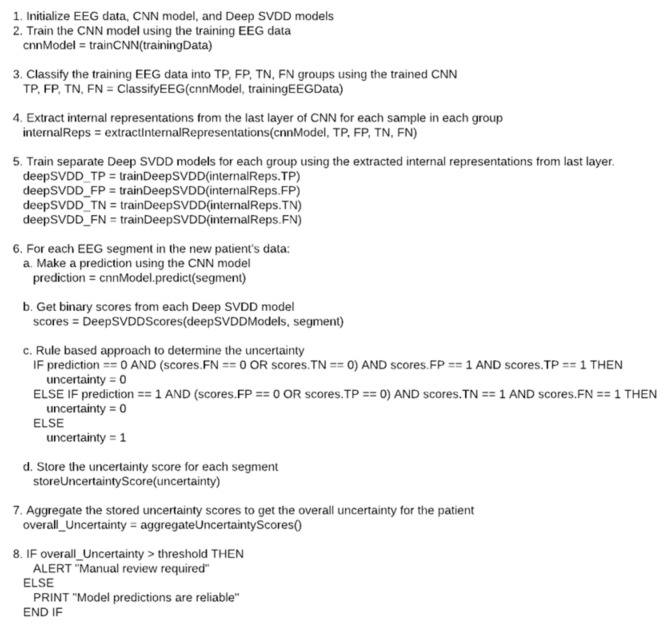
Shows the pseudocode for the process for uncertainty estimation based on a patient.

On the other hand, if the prediction is ‘1′ (seizure) and either the FP or TP group prediction is 0, while the TN and FN predictions are both ‘1′, it implies that the model is likely confident in its seizure prediction. If the predictions do not meet any of the conditions mentioned above, the model is considered uncertain about its predictions. The Pseudocode for the whole process is displayed in Figure 3.

Ultimately, each EEG segment yields a binary label along with its prediction, where 0 indicates confidence and ‘1′ signifies uncertainty. These binary labels are then aggregated by averaging to produce a probability score ranging from ‘0–1′. This score represents the model’s overall level of uncertainty in its predictions for the patient data, with ‘0′ signifying total confidence and 1 indicating complete uncertainty.

### 3.4. Classification Model for Seizure Detection

We employed the CNN as our DL model used for training as well as prediction. CNN models have traditionally been employed for image recognition tasks, where they excel in automatically learning temporal, spatial, and spectral features from inputs, eliminating the need for traditional feature extraction or engineering. Moreover, the use of CNN models in seizure detection has been documented in numerous studies, consistently demonstrating superior performance on publicly accessible datasets, with an achieved accuracy exceeding 90%. In our study, we will use this model to demonstrate the ability of our proposed method to detect patients that the model is uncertain of.

**Figure 4:**
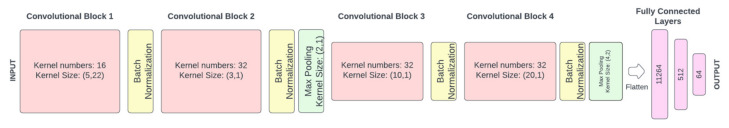
Shows the CNN architecture of our DL model.

Our tailored CNN model is composed of an input layer, multiple hidden layers and an output layer. We have represented electroencephalogram (EEG) signals as an image-like matrix, utilizing channels (C) and time (T) as inputs. The input representation for our model adheres to an N × C × T × D structure, with N indicating the number of samples, C symbolizing channels, T representing time and D standing for dimension. The architecture of our model incorporates four fully connected CNN layers. In order to extract spatial features between channels, the 1st convolutional block extracts features across channels. This is achieved through a kernel size of (5, 2), helping to capture inter-relationships between channels. The remaining 3 blocks of convolutional layers extract temporal features within each of the channels with varying time scales, from short term (3, 1) to long term (20, 1). This is because short-term kernels are able to capture abrupt or rapid changes in the EEG signals, that could be an indication of seizures with a high frequency of amplitude. Longer-term kernels are able to capture general wave form, rhythmic or periodic seizure patterns. To mitigate overfitting and computational complexity, we employed multiple pooling and dropout layers. The features are then flattened to 1 dimension for input into fully connected layers for predictions. Our model was developed using the Pytorch 2.0 framework, implemented using Python 3.8. A detailed overview of the architecture implementation is presented in Figure 4.

We conducted the training process utilizing the NVIDIA RTX 3080 GPU (Nvidia Corporation, Santa Clara, CA, USA), with 100 epochs, a learning rate set at 0.0001 and a batch size of 32. The training duration for each patient was approximately two hours. We utilized the Leave One Patient Out Cross Validation (LOPO-CV) method to train and validate our CNN model’s performance. LOPO-CV is commonly employed in the field of seizure detection to evaluate a machine learning models’ performance and involves leaving out one patient for testing, while the rest are used for training. The test set remains unseen by the model during the training process. This procedure is repeated for all 23 patients, and the performance of the model is subsequently averaged across all patients. In contrast, typical K-fold cross-validation (KFCV) often combines data from all patients into one dataset, leading to potential data leakage if a patient’s data appears in both the training and test sets. This can result in unreliable predictions and false results. LOPO-CV effectively prevents this issue. This approach of training on existing patients and testing on unseen patients aligns with real-world scenarios where it needs to predict seizure events of previously unencountered patients.

Our CNN model demonstrated an average Area Under the Curve (AUC) of 91%, an accuracy of 92%, a specificity of 92%, sensitivity of 78% and F1 score of 71% with Standard Deviations (SDs) of 0.12, 0.09, 0.09, 0.20 and 0.21, respectively, across all patients. When compared to state-of-the-art CNN models that utilized similar minimal feature engineering and preprocessing techniques, our model displayed comparable performance. The results for the performance of each patient can be found in Table 1.

## 4. Evaluation

To evaluate the effectiveness of our proposed methods in identifying patients for whom the model is uncertain, we compare our model with other commonly used techniques that can be used to detect uncertainty in a patient’s data such as SoftMax confidence, Deep SVDD, and MC Dropout. Similar to our proposed method, we aggregated the binary labels generated by these methods to produce a probability score of between 0 and 1 uncertainty.

The effectiveness of our technique in identifying uncertainty in patients is tested by generating truth labels, which are based on each patient’s performance through the CNN models. These truth labels act as a measure of the model’s confidence, designated as confident if the F1 score exceeds a threshold of 0.5, implying that the model’s performance exceeds chance levels for that specific patient. Conversely, an F1 score falling below this threshold indicates that the model’s performance was suboptimal for the patient in question.

To simulate a more conservative application of the DL model, akin to the cautious approach often taken by clinicians, we raise the F1 score threshold to 0.7, while keeping the conditions consistent with the previously described scenario. This change in threshold reflects a higher level of confidence required for the model’s performance to be considered effective for each individual patient.

First, we examine if there is a correlation between the F1 score of the model and the uncertainty levels produced by each of the methods by calculating the Pearson Correlation Coefficient (r). This analysis allows us to understand how the uncertainty score relates to the model’s performance in detecting seizures (sensitivity) in patients. An effective uncertainty estimation technique is defined when there is an inverse correlation between the F1-score of the model and the uncertainty level produced by the technique.

Second, we assess the method’s ability to identify patients for which it is confident or uncertain in its predictions. We use the aggregated uncertainty score produced by each method for this purpose. If the aggregated score produced by each method is below 0.5, it indicates that the model is confident in its prediction; conversely, a value above 0.5 indicates that the model is uncertain about the prediction it has made.

## 5. Results

**Figure 5:**
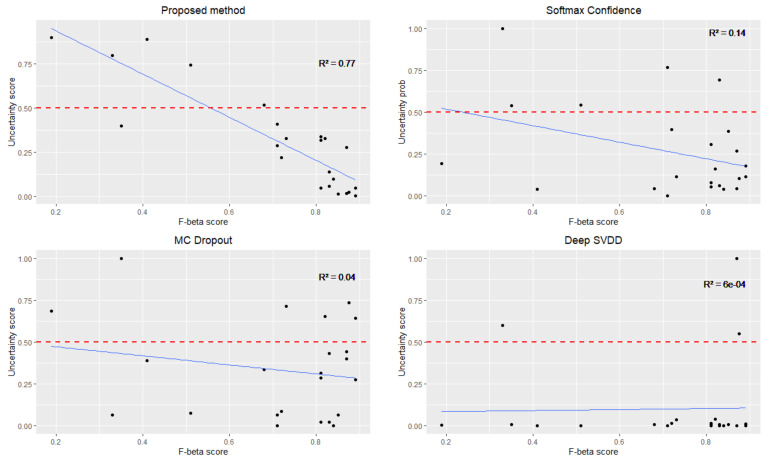
Shows each of the methods for detecting uncertainties in patients (block dots), indicating unreliable predictions for the patients if it falls below the threshold at 0.5 uncertainty score (red dotted line). Blue line indicates relationship between uncertainty score and F-beta score.

As indicated in Table 2, our proposed method has the strongest correlation coefficient of −0.88, compared to −0.37, 0.19 and 0.02 for SoftMax confidence, MC Dropout and Deep SVDD, respectively. This indicates a strong negative correlation between the uncertainty value yielded by our proposed model and the F1-score produced by the CNN model as seen in Figure 5. As the model’s F1-score improves, our uncertainty value tends to decrease, signifying greater confidence in the model’s predictions. In comparison with other methods, none of the commonly used methods produced any meaningful correlations. The SoftMax confidence shows a weak negative correlation, while MC Dropout exhibits a very weak negative correlation. Deep SVDD shows no linear correlation at all.

Based on Table 3, our proposed method achieved an accuracy of 0.89 in correctly classifying patients that it is confident and uncertain of. Out of the five patients whose CNN model is unable to perform well (F1-score < 0.5), the proposed method can detect three patients for whom the CNN model might not be able to detect a seizure well (75% sensitivity). Our method accurately indicated confidence in the model’s predictions for nearly all cases where the model was indeed confident, displaying a specificity of 89%.

In comparison to our methods, the other methods were largely ineffective in detecting uncertainty in most patients, all showing a sensitivity of 50% or lower. Because the uncertainty scores produced by SoftMax confidence and MC Dropout were small, we attempted to rescale the uncertainty scores using Min-Max scaling, so that the maximum uncertainty score for each method would be 1. This step was undertaken to enhance comparability across different methods.

In circumstances where clinicians take a more conservative approach, necessitating a higher sensitivity with an F1-score threshold of 0.7, our proposed method continues to outperform other techniques. It demonstrates an enhanced performance across all measures, achieving an overall accuracy of 0.96 and a sensitivity of 0.83, as seen in Table 4. When we assume an extremely conservative approach, with threshold at 0.8, our performance still outperformed the other methods, but the sensitivity in detecting uncertainty patients dropped to 0.5.

## 6. Discussions

Traditional methods, such as Deep SVDD or OOD, often fall short as they do not adequately consider the intricate nature of EEG data. These conventional approaches are limited in capturing the complex, nonlinear dynamics inherent in distinguishing between seizure and non-seizure events. CNN models occasionally offer high-confidence predictions that may not always be accurate, especially in the context of seizure events. This overconfidence undermines the reliability of methods that solely rely on prediction probabilities or techniques like MC Dropout. For instance, our CNN models often emit incorrect high-confidence, one-sided probability predictions for seizure events, even when faced with unseen data from a patient. This scenario underscores the model’s unreliability and the need for more advanced, nuanced uncertainty estimation techniques. Furthermore, a single SVDD might also encounter significant limitations in adequately processing complex and variable EEG patterns, where they often look similar to untrained eyes. Similarly, the single model provides a too-simplified analysis, insufficient for accurately identifying and discriminating between the seen and unseen patterns. Its primary shortfall lies in its inability to fully comprehend the diverse nature of EEG data, resulting in an often-imprecise estimation of uncertainty and identification of out-of-distribution (OOD) data.

In this study, our proposed solution of deploying individual Deep SVDD models for each data group along with a simple rule-based approach addresses this limitation. Each model is tailored to learn and discern patterns specific to its assigned group effectively. This adaptation caters to the variances in learned seizure patterns and seizure patterns that it fails to learn, enhancing the overall accuracy and reliability of uncertainty estimations. The proposed method was assessed for 23 patients and validated based on the performance of the predictions made by the CNN model. This represents the first attempt in the field of seizure detection to introduce uncertainty in data at the patient level.

We recognize that our CNN model does not surpass the latest state-of-the-art models. However, our study aims to identify patients for whom the model’s predictions are uncertain. Another reason for choosing the CNN model is that it encompasses a mix of results for patients with good and poor performance, enabling us to test our algorithm on both sets of patients.

Our proposed technique for detecting uncertainty in input data for a given patient outperforms commonly used methods when applied to our scenario. Our method enhances the safety of seizure detection algorithms by effectively identifying patients where the seizure detection algorithms do not perform well in detecting seizures, achieving a sensitivity of 75%. This high sensitivity rate is beneficial in preventing false negatives, which could have serious consequences for patient care. We also hypothesized that the poor performance of conventional methods could be due to the model’s inherent nature of producing high-confidence predictions even if the EEG data of the patients has not been seen in the training set, where they have no ability to discriminate between unseen and seen EEG recordings.

The proposed method is independent of the model choice and can be adapted and applied to most DL algorithms, making it a flexible and versatile solution in identifying uncertainty in patients. It also performs well on imbalanced datasets, which is often the case for seizure detection EEG datasets, compared to other methods during evaluation. This robustness ensures a reliable performance, even in challenging situations, and helps to improve the overall quality of the predictions by eliminating patients where the model was not confident in the predictions.

Since our proposed technique for detecting uncertainty is based on the data of a patient and only learns from their own training distribution using an unsupervised learning method, it does not require any testing labels. This simplifies the process since it does not require any additional unseen data for learning, reducing the need for extensive data collection and preprocessing. As the proposed technique learns from its own training patterns, it is particularly useful when the dataset is limited, which is a common challenge in EEG data acquisition.

Our proposed technique allows for further customization to suit the risk appetite of users. For example, if the threshold for the uncertainty percentage is set to 40%, which indicates a clinician with a low risk tolerance, the algorithm successfully detects all five patients where the model yields a low F1-score (below 0.7). However, it might exclude patients for whom the model predicts reasonably well, as in the case of patient 8, who scores 0.71 on the F1-score with an uncertainty value of 0.41. This adaptability enables clinicians to tailor the model’s performance to their specific needs and risk tolerance, ensuring optimal patient care and resource allocation.

The proposed technique aims to reduce the level of uncertainty in its predictions at the patient level, rather than focusing solely on individual data segments. By enhancing the confidence in the predictions made for a patient as a whole, the model can help improve the overall reliability of the assessment for that patient. Our model is designed to minimize uncertainty in predictions at the patient level, with the aim of eliminating situations where the model has no confidence in its assessments. By concentrating on decreasing uncertainty for each patient as a whole, the model can significantly enhance the overall reliability of evaluations. While this strategy does not assure absolute accuracy or certainty, its primary objective is to identify and mitigate risks associated with low-confidence predictions for each patient.

## 7. Limitations

While the method proposed here helps to improve the confidence of the model given the data of a patient, it has a few limitations. Our model was only tested on 23 patients and has not been validated with other publicly available datasets. Moving forwards, we plan to validate our performance in other EEG datasets to evaluate the effectiveness of our proposed method.

Moreover, our technique incorporates Deep SVDD as part of the process. In situations where the training data for groups, such as TP or FP, is limited, the model may overfit. This could generate unreliable results due to its high specificity to the training data, potentially hampering its ability to generalize effectively with new, unseen data. This overfitting issue, stemming from limited training data in certain groups, also makes the model prone to volatility, leading to unstable results. Further research and fine-tuning of hyperparameters, along with exploration of more robust sampling techniques, are needed to enhance generalizability when dealing with small training data sets.

Lastly, our method introduces additional computational complexity as it necessitates the training of four more neural networks. This is especially relevant for large-scale datasets and may limit the practicality of our method in scenarios where computational resources are limited.

## 8. Conclusions

In conclusion, this study introduces a novel approach to detecting uncertainty at the patient level in seizure detection by incorporating methods from OOD and uncertainty estimation. Our method successfully identifies most patients for whom the model fails to detect seizures effectively. This approach not only streamlines the annotation process by alerting clinicians to patients where the algorithm might fail to accurately detect seizures in certain patients, but it also helps identify patients who may require more manual attention from healthcare professionals. Ultimately, our method aims to enhance the overall efficiency and effectiveness of seizure detection while ensuring that clinicians can provide targeted and informed care for each patient.

## Figures and Tables

**Table 1:** Shows the performance of the trained CNN model for each model.

Patient	Accuracy	Specificity	Sensitivity	AUC	F1 Score
1	0.95	0.95	0.95	0.99	0.89
2	0.97	0.97	0.91	0.98	0.83
3	0.92	0.92	0.9	0.95	0.81
4	0.93	0.93	0.81	0.93	0.71
5	0.89	0.89	0.94	0.97	0.83
6	0.92	0.92	0.31	0.74	0.19
7	0.98	0.98	0.87	0.98	0.81
8	0.88	0.88	0.74	0.89	0.71
9	0.98	0.98	0.95	0.98	0.87
10	0.95	0.95	0.94	0.97	0.87
11	0.95	0.95	0.91	0.98	0.89
12	0.94	0.94	0.83	0.95	0.82
13	0.54	0.55	0.41	0.46	0.35
14	0.97	0.97	0.73	0.95	0.68
15	0.91	0.91	0.34	0.81	0.33
16	0.95	0.95	0.53	0.9	0.41
17	0.85	0.85	0.83	0.93	0.72
18	0.96	0.96	0.87	0.96	0.81
19	0.98	0.98	0.88	0.98	0.84
20	0.93	0.93	0.57	0.79	0.51
21	0.94	0.94	0.84	0.96	0.73
22	0.94	0.94	1	0.99	0.875
23	0.93	0.93	0.91	0.97	0.85
Average ± SD	0.92 ± 0.09	0.92 ± 0.09	0.78 ± 0.20	0.91 ± 0.12	0.71 ± 0.21

**Table 2:** Shows the Pearson Correlation coefficient for each of the proposed methods, comparing the uncertainty score and the F1-Score.

Technique	Pearson Correlation Coefficient (r)
Proposed Method	−0.88
SoftMax Uncertainty	−0.37
MC Dropout	−0.19
Deep SVDD	0.02

**Table 3:** Shows the performance of each method for uncertainty estimation in 23 patients, with threshold of 0.5.

	Accuracy	Specificity	Sensitivity	AUC
Proposed Method	0.89	0.94	0.75	0.96
SoftMax confidence	0.78	0.84	0.50	0.64
MC Dropout	0.74	0.79	0.50	0.70
Deep SVDD	0.78	0.89	0.25	0.54

**Table 4:** Shows the performance of each method of uncertainty estimation in 23 patients, with conservative thresholds of 0.7 and 0.8.

F1 Threshold	0.7				0.8			
Metrics	Accuracy	Specificity	Sensitivity	AUC	Accuracy	Specificity	Sensitivity	AUC
Proposed Method	0.96	1	0.83	0.99	0.78	1	0.5	0.99
SoftMax confidence	0.78	0.88	0.5	0.61	0.69	0.92	0.4	0.61
MC Dropout	0.65	0.76	0.3	0.62	0.52	0.75	0.3	0.62
Deep SVDD	0.7	0.88	0.17	0.44	0.43	0.82	0.11	0.44

## Data Availability

Not applicable.
